# Recombinant human IL-37 inhibited endometriosis development in a mouse model through increasing Th1/Th2 ratio by inducing the maturation of dendritic cells

**DOI:** 10.1186/s12958-021-00811-3

**Published:** 2021-08-24

**Authors:** Lijie Li, Zhouzhou Liao, Mingzhu Ye, Jianfa Jiang

**Affiliations:** Department of Gynecology, The Third Xiangya Hospital of Central South University, NO.138 tongzipo, Yuelu District, Hunan 410013 Changsha, China

**Keywords:** Endometriosis, Recombinant human IL-37, Dendritic cell, T cell differentiation

## Abstract

**Background:**

Endometriosis is a serious reproductive and general health consequences. Recombinant human IL-37 (rhIL-37) is an inhibitor of inflammation.

**Methods:**

ELISA assay was performed to detect the concentration of cytokines. Flow cytometry was used to analyze cell proportion. Besides, qRT-PCR and western blotting assay were used to detect the level of gene and protein, respectively. Transwell co-culture system was used for the co-culture of dendritic cells (DCs) and CD4^+^T cells.

**Results:**

Our data showed that rhIL-37 inhibited the development of ectopic lesions in the mice with endometriosis, increased Th1/Th2 ratio and induced DCs maturation. The co-culture system of DCs and CD4^+^T cells demonstrated that rhIL-37 increased Th1/Th2 cell ratio through promoting DCs maturation. Moreover, the expression of IL-4 in the DCs derived from healthy mice was inhibited by rhIL-37 treatment. rhIL-37 increased Th1/Th2 cell ratio through inhibiting IL-4 in DCs. Subsequently, our results proved that rhIL-37 promoted the maturation of DCs via inhibiting phosphorylation of STAT3. Activation of STAT3 could reverse rhIL-37-induced maturation of DCs.

**Conclusion:**

Overall, rhIL-37 could protect against endometriosis through increasing the ratio of Th1/Th2 cells via inducing DCs maturation and inhibiting IL-4 expression in the DCs. Furthermore, rhIL-37 induced DCs maturation by inhibiting STAT3 phosphorylation. Our data confirmed the protective effect of rhIL-37 in endometriosis. These data may provide a novel idea for the treatment of the disease.

**Graphical abstract:**

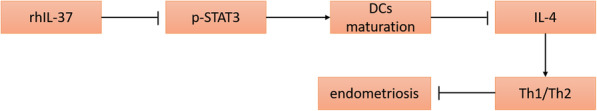

**Supplementary Information:**

The online version contains supplementary material available at 10.1186/s12958-021-00811-3.

## Background

In clinical, endometriosis (EMs) is a common gynecological disease characterized by activated endometrial cells plant onto the outside of endometrium [1]. The incidence of endometriosis in women of childbearing age is about 10% ~ 15%, but the incidence of it is up to 30% in the patients with infertility or chronic pelvic pain [2]. Endometriosis is a serious reproductive and general health consequences. Importantly, it was reported that the patients with endometriosis have a higher risk of developing ovarian cancer [3]. Currently, the goals of endometriosis treatment are to reduce operative intervention, fertility preservation, prevent disease recurrence, improve the quality of life, and pain control. Although some drugs, such as GnRH antagonists, aromatase inhibitors, and antiprogestins, could effectively protect against endometriosis, the treatment of the disease still is a challenge [4, 5]. It is very necessary to explore the pathogenesis of endometriosis, and explore a novel idea for the treatment of the disease.

It was well known that endometriosis is a choric and inflammatory disease [6]. The concentration of pro-inflammatory cytokines like tumor necrosis factor-α (TNF-α) was highly expressed in the peritoneal fluid of the patients with endometriosis [7]. Abnormal immune system is closely associated with the development of endometriosis. The number of immune cells was obviously increased in the serum and peritoneal fluid of the patients with endometriosis, and the proportions of T helper (Th) cells (Th1 and Th2 cells) were imbalanced in the serum of patients [8, 9]. It was reported that the concentration of Th1 cell-related cytokines like interferon-γ (IFN-γ) was lowly expressed, while Th2 cell-related cytokines like interleukins (IL)-4, IL-10, and IL-13 were highly expressed in the serum of the patients with endometriosis [10, 11]. Moreover, dendritic cells (DCs) also play a crucial role in the development of endometriosis. DCs are specialized immune cells, which involve in both innate and adaptive T cells-mediated immunological responses [12]. It was demonstrated that the number of immature dendritic cells (iDCs) was notably higher than mature dendritic cells (mDCs) in the endometriosis tissues from a non-human primate model of the disease [13]. In previous study, Fainaru et al. revealed that immature bone marrow-derived DCs, not mature bone marrow-derived DCs, contribute to the development of endometriosis [14]. These studies suggested the important role of mDCs in the improvement of endometriosis.

IL-37 is a unique member of the IL-1 family, and participates in the development of multiple diseases, for example, colitis, arthritis, sepsis, and endotoxin shock. IL-37 is a natural suppressor of inflammatory, and play a protective role in above diseases [15, 16]. Recently, some studies demonstrated that IL-37 could suppress the production of pro-inflammatory cytokines like IL-1β, IL-6, and IL-10, and inhibit the occurrence and development of endometriosis through targeting multiple signaling pathways, such as mitogen-activated protein kinase signaling and Wnt/β-catenin [17]. He et al. indicated that IL-37b splice variant could effectively suppress the growth of lesion in an endometriotic mouse model through regulating the invasion, angiogenesis, proliferation and inflammation by affecting AKT and ERK1/2 signaling [18]. However, the effect of IL-37 on the abnormal immune cell remains unclear. Here, in our present study, the data revealed that recombinant human IL-37 (rhIL-37) could inhibit the development of endometriosis through increasing the ratio of Th1/Th2 cells. Mechanismly, rhIL-37 increased Th1/Th2 ratio through inducing the maturation of DCs and inhibiting IL-4 production via suppressing the phosphorylation of signal transducer and activator of transcription 3 (STAT3). Our study proved again the therapeutic ability of rhIL-37 in endometriosis, and may provide a novel idea for the treatment of endometriosis.

## Materials and methods

### Reagents

Female C57BL/6 mice (6–8 weeks; 19–24 g) were obtained from the Jiangsu Ailingfei Biotechnology Co., Ltd. (Nanjing, China). Here, rhIL-37 was obtained from Abcam (ab151873, USA). Estradiol benzoate (E8875-250MG), lipopolysaccharide (LPS, SMB00704), and IL-4 (SRP3093) were purchased from Sigma-Aldrich (California, USA). Colivelin, an activator of STAT3, was bought from Santa Cruz Biotechnology, Inc. (CAS 867021–83-8, Dallas, TX, USA). FITC-labeled anti-CD4 (11–0048-42), APC-labeled anti-CD11c (11–0116-42), APC-labeled anti-interferon-γ (IFN-γ, 17–7319-82), PE-labeled anti-IL-4 (Th2 cells, 12–7049-42), PE-labeled anti-CCR5 (12–1956-42), PE-labeled anti-CD83 (12–0839-42), Biotin-labeled anti-MHC II (MA1–12180), FITC-labeled anti-CD40 (11–0409-42), PE-labeled anti-CD80 (12–0809-42), APC-labeled anti-CD86 (MHCD8605) were purchased from eBioscience (California, USA). RPMI-1640 medium (11875119), fetal bovine serum (10100147), and penicillin-streptomycin sulfate (15140148) were obtained from Gibco (USA). The Transwell-6 co-culture system with a 0.4 μm porous membrane was bought from Corning (NY, USA). Moreover, the ELISA kits, including mouse IL-37 ELISA kit (ml058377), mouse IFN-γ ELISA kit (ml058350-J), mouse TNF-α ELISA kit (ml002095), mouse IL-4 ELISA kit (ml063156-J), and mouse IL-13 ELISA kit (ml063123), were purchased from Shanghai Enzyme-linked Biotechnology Co., Ltd. (Shanghai, China). TRIzol reagent was from Invitrogen (Carlsbad, CA, USA). Transcriptor First Strand cDNA Synthesis Kit was from Roche (04379012001, Basel, Switzerland). SYBR Premix Ex Taq was from Takara (Dalian, China). RIPA lysis buffer (R0010), BCA Protein Assay Reagent Kit (PC0020), and enhanced chemiluminescence kit (PE0010) were obtained from Solarbio (Shanghai, China). The primary antibodies like anti-STAT3 (ab68153) and anti-p-STAT3 (ab267373), and the secondary goat anti-rabbit (ab6721) were purchased from Abcam (Cambridge, MA, USA).

### Establishment of endometriosis mouse model

All mice were housed in a standard environment with 60–70% relative humidity, 22–24 °C of temperature, and 12 h light/12 h dark cycle. At 1 week after adapt, 3 μg of estradiol benzoate dissolved in 50 μl of soybean oil was subcutaneously injected into each donor mouse. After 1 week of estradiol benzoate injection, the uteri tissues of donor mice were dissected, and then the endometrial tissues were obtained. After that, all endometrial tissues were cut into < 1 mm^3^ pieces. These pieces were suspended in normal saline. The pieces from one mouse was suspended in 1 ml normal saline. Subsequently, each recipient mouse were administrated with 500 μl of tissues suspension. Then, the recipient mice were randomly assigned to different groups. All animal experiments were approved by the ethical committee of the Third Xiangya Hospital of Central South University (No. 2018-S146), and performed strictly in accordance with animal experiment guidelines and regulations in the Third Xiangya Hospital of Central South University.

### Isolation of CD4^+^T cells and DCs, and cell culture and treatment

Peritoneal lavage fluid samples were obtained from healthy mice and the mice with endometriosis at the time of sacrifice through peritoneal lavage with 5 ml of ice-cold PBS. Then, the peritoneal lavage fluid samples were centrifuged at 1500 g for 5 min to obtain the cells of intraperitoneal lavage. Cells were subsequently resuspended in PBS, and were stained with FITC-labeled anti-CD4 (CD4^+^T cells), or APC-labeled anti-CD11c (DCs) antibodies in the dark for 30 min at 4 °C. Subsequently, a flow cytometer (FACSVerse, BD, New York, USA) was utilized to separate CD4^+^T cells from the intraperitoneal lavage of healthy mice, and isolate DCs from the intraperitoneal lavage of healthy mice (control-DCs) and endometriosis mouse model (EMs-DCs).

CD4^+^T cells were cultured in the RPMI-1640 medium supplemented with 10% fetal bovine serum, 100 U/mL penicillin and 100 μg/mL streptomycin sulfate. DCs were cultured in the RPMI-1640 medium supplemented with 10% fetal bovine serum, 100 U/mL penicillin, 100 μg/mL streptomycin sulfate, 10 ng/ml IL-4, and 20 ng/ml recombinant mouse GM-CSF. All cells were grown at 37 °C in a humidified atmosphere with 5% CO_2_. For the different cellular experiments, 100 ng/mL rhIL-37 was used to stimulate DCs, 100 ng/mL LPS was utilized to induce the maturation of iDCs, and 100 ng/mL IL-4 was used to stimulate DCs. Besides, 0.5 μM Colivelin was chosen as the STAT3 activator.

### Administration of rhIL-37 to mouse

For animal experiments, the endometriosis mouse model was randomly divided into three groups: EMs, EMs + NS, and EMs + rhIL-37. A total of six model mice of endometriosis were assigned to the EMs group. Besides, 12 mice with endometriosis were balanced distributed to EMs + NS and EMs + rhIL-37 groups. The mice in EMs + NS group were intraperitoneally injected with normal saline at 24 h before modeling. The mice in EMs + rhIL-37 group were intraperitoneally injected with rhIL-37 which was dissolved into normal saline, for 1000 ng per mouse at 24 h before modeling. The administration of normal saline and rhIL-37 was performed for once every 2 days, and was performed for a total of 10 times. Besides, the C57BL/6 mice in control group were suffered from acupuncture, but were injected with nothing. At 24 h after the last normal saline and rhIL-37 injection, all mice were sacrificed through cervical dislocation. Next, the ectopic lesions were observed, and the weight and volume of tissues were evaluated. Meantime, the peripheral blood samples of each mice were obtained for next experiments. The drug administration, sampling, and sacrifice process to the mice was shown in Supplementary Fig. 7.

### Detection of IL-37, IFN-γ, TNF-α, IL-4, and IL-13

The concentrations of IL-37, IFN-γ, TNF-α, IL-4, and IL-13 in serum, and the production of IL-4 in DCs were measured by ELISA assay. All experiment were carried out strictly in accordance with the manufacture’s introductions of the mouse IL-37 ELISA kit, mouse IFN-γ ELISA kit, mouse TNF-α ELISA kit, mouse IL-4 ELISA kit, and mouse IL-13 ELISA kit. The OD values at 450 nm of each well were examined utilizing a microplate reader (BioRad Model 680, USA), and the reading results were saved in the instrument.

### Analysis of Th1/Th2 cells ratio, iDCs and mDCs percentages, and MHC II-, CD40-, CD80-, and CD86-positive DCs cells

Flow cytometry was carried out to analyze the proportions of Th1, Th2, iDCs, and mDCs, and the expression of MHC II, CD40, CD80, and CD86 in the surface of DCs cells. In order to detect the differentiation of Th1 and Th2 cell, the CD4^+^T cells were stained with APC-labeled anti-IFN-γ (IFN-γ^+^T cells, Th1 cells) and PE-labeled anti-IL-4 (IL-4^+^T cells, Th2 cells). In order to analyze the maturation of DCs, the DCs were stained with APC-labeled anti-CD11c and PE-labeled anti-CCR5 (CD11c^+^CCR5^+^DCs, iDCs), or APC-labeled anti-CD11c and PE-labeled anti-CD83 (CD11c^+^CD83^+^DCs, mDCs). Moreover, DCs were stained with PE-labeled anti-MHC II, FITC-labeled anti-CD40, PE-labeled anti-CD80, and APC-labeled anti-CD86. During the process, the cells were incubated with above antibodies in the dark for 30 min at 4 °C. Finally, the percentages of the Th1, Th2, iDCs and mDCs, and the percentages of MHC II-, CD40-, CD80-, and CD86-positive cells were analyzed utilizing a flow cytometer (FACS Aria; BD) with a Flow Jo v10.0.7 software.

### Co-Culture system of CD4^+^T cells and DCs

The co-culture system of CD4^+^T cells and DCs was conducted using a Transwell system. CD4^+^T cells were planted into the upper chamber of Transwell chamber, and DCs were seeded into the bottom chamber. CD4^+^T cells were co-treated with LPS, rhIL-37 and IL-4 for 24 h. Subsequently, the maturation of DCs, ratio of Th1 and Th2 cells, and expression of molecules were measured.

### Measurement of genes expression

The expression levels of IFN-γ mRNA, TNF-α mRNA, IL-4 mRNA, and IL-13 mRNA were measured by qRT-PCR. Total RNA was isolated from CD4^+^T cells using TRIzol reagent. Then, the total RNA served as the template in reverse transcription, which was carried out according to the manufacture’s protocol of the Transcriptor First Strand cDNA Synthesis Kit. Subsequently, real-time PCR was performed on an ABI 7500 Real-time PCR system (Applied Biosystems, Foster City, CA, USA) using the SYBR Premix Ex Taq. The relative expression levels of IFN-γ mRNA, TNF-α mRNA, IL-4 mRNA, and IL-13 mRNA were normalized to *GAPDH*, and were calculated in accordance with 2^-ΔΔCt^ method. The gene sequence of primers were as follows: IFN-γ: 5′-CTTCTTCAGCAACAGCAAGG-3′ (F) and 5′-TGAGCT CATTGAATGCTTGG-3′ (R); TNF-α: 5′-GCTCTTCTGTCTACTGAACTTCGG-3′ (F) and 5′-ATGATCTGAGTGTGAGGGTCTGG-3′ (R); IL-4: 5′-CACAACTGAGA AGGAAACCTTCTG-3′ (F) and 5′-CTCTCTCATGATCGTCTTTAGCCTTTC-3′ (R); IL-13: 5′-GCTCCTCAATCCTCTCCTGTT-3′ (F) and 5′-GCAACTTCAATAGTCAG GTCC-3′; GAPDH: 5′-TCCACCACCCTGTTGCTGTA-3′ (F) and 5′-ACCACAGTC CATGCCATCAC-3′ (R).

### Detection of the expression of STAT3 and its phosphorylation

The expression levels of STAT3 and p-STAT3 were measured using western blotting assay. Total protein was separated from DCs using RIPA lysis buffer. Then, the concentration of protein was determined using a BCA Protein Assay Reagent Kit. After that, 25 μg of protein was separated on 12% SDS-PAGE gel, and were transferred onto PVDF membranes. The membranes were then maintained with 5% non-fat milk for 1 h at room temperature followed by the anti-STAT3 and anti-p-STAT3 antibodies incubation overnight at 4 °C. Next day, the membranes were incubated with secondary goat anti-rabbit for 1 h at room temperature. At last, an enhanced chemiluminescence kit was utilized to determine the protein bands, and the optical density of the western blot was analyzed using the Image-Pro Plus 6.0 (Media Cybernetics, lnc., USA) software. The relative expression of STAT3 and p-STAT3 was normalized to β-actin.

### Statistical analysis

SPSS 19.0 (SPSS Inc., USA) software was utilized for all data analysis, which was displayed as mean ± standard deviation (SD). The statistical difference among multiple groups were determined using one-way analysis of variance (ANOVA) followed by Bonferroni’s test. The statistical difference between two independent groups were determined by Student’s *t*-test. The value of *P* lower than 0.05 was recognized as statistically significant. All experiments were independently repeated for three times at least.

## Results

### rhIL-37 inhibited lesion development, increased serum Th1/Th2 ratio, and induced DCs maturation in the mice with endometriosis

**Fig. 1:**
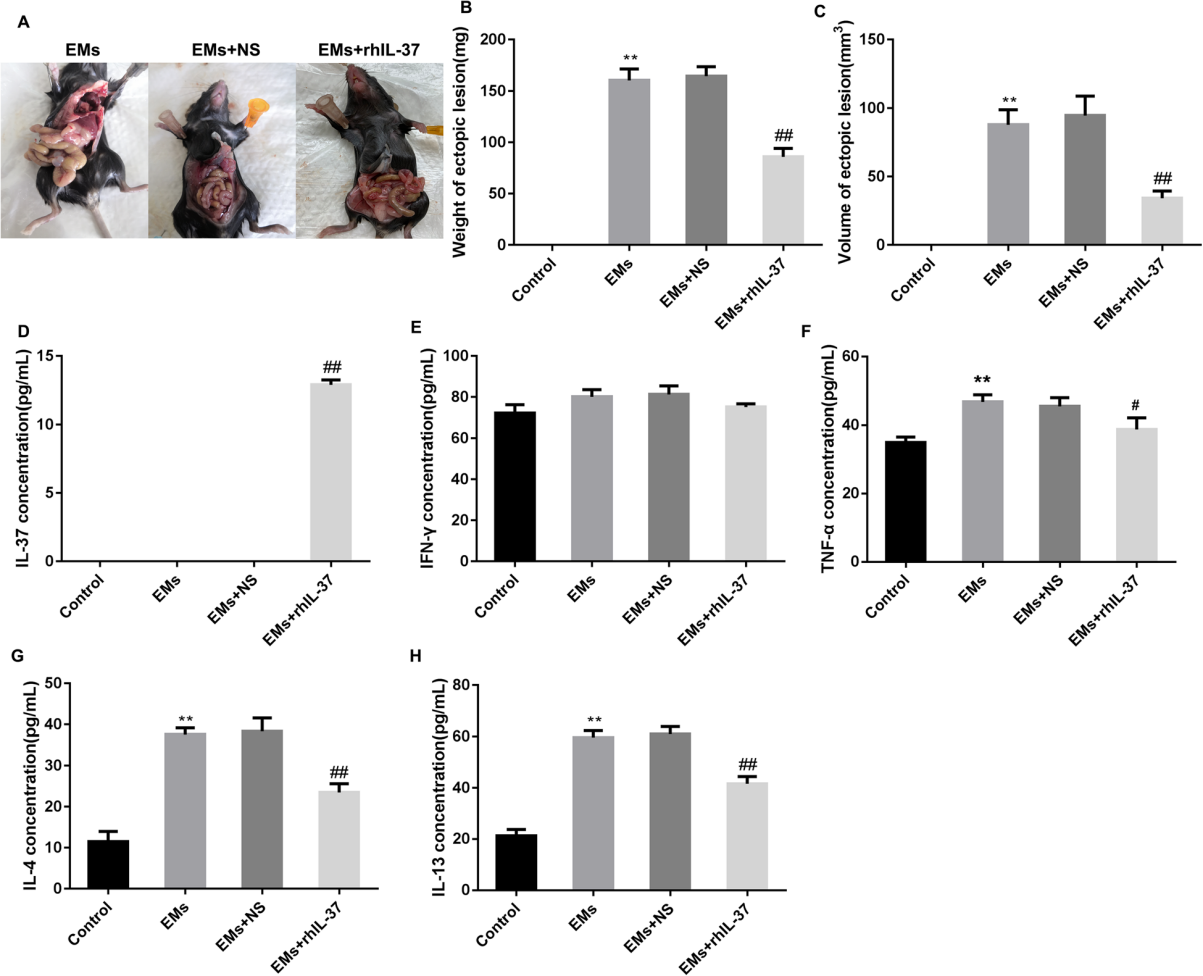
rhIL-37 inhibited the development of lesion in the endometriosis mouse model. At 24 h after the last rhIL-37 administration, (**A**) the formation of ectopic lesions were observed; (**B**-**C**) the weight and volume of ectopic lesions were measured; (**D**-**H**) the serum IL-37, IFN-γ, TNF-α, IL-4, and IL-13 levels were measured using ELISA assay. *N =* 6. **P < 0.01 compared with Control, and ^##^*P* < 0.01 compared with EMs

Here, compared with the mouse with endometriosis and normal saline-treated endometriosis mouse model, declined weight of ectopic lesion and reduced volume of ectopic lesions were found in the rhIL-37-treated endometriosis mouse model, suggesting that rh-IL-37 treatment effectively inhibited the development of ectopic lesions (Fig. 1A-C). ELISA assay displayed that rhIL-37 was highly existed in the serum of the mice with endometriosis, and no rhIL-37 was found in the serum of control mice, endometriosis mouse model, and normal saline-treated endometriosis mouse model (Fig. 1D). Interestingly, for the mice in Control, EMs, EMs + NS, and EMs + rhIL-37 groups, there was no significant difference in the level of serum IFN-γ (Fig. 1E). Nevertheless, the production of serum TNF-α, a pro-inflammatory cytokine, was notably upregulated in the mice with endometriosis, which was partly downregulated by rhIL-37 treatment (Fig. 1F). Besides, the levels of serum IL-4 and IL-13 were upregulated in the mice with endometriosis, but rhIL-37 treatment could effectively decline the levels of them (Fig. 1G and H). IFN-γ, TNF-α, IL-4, and IL-13 are the important cytokines for Th1 and Th2 cells, hence, above results indicated that rhIL-37 maybe improve endometriosis through regulating Th1 and Th2 differentiation.

**Fig. 2:**
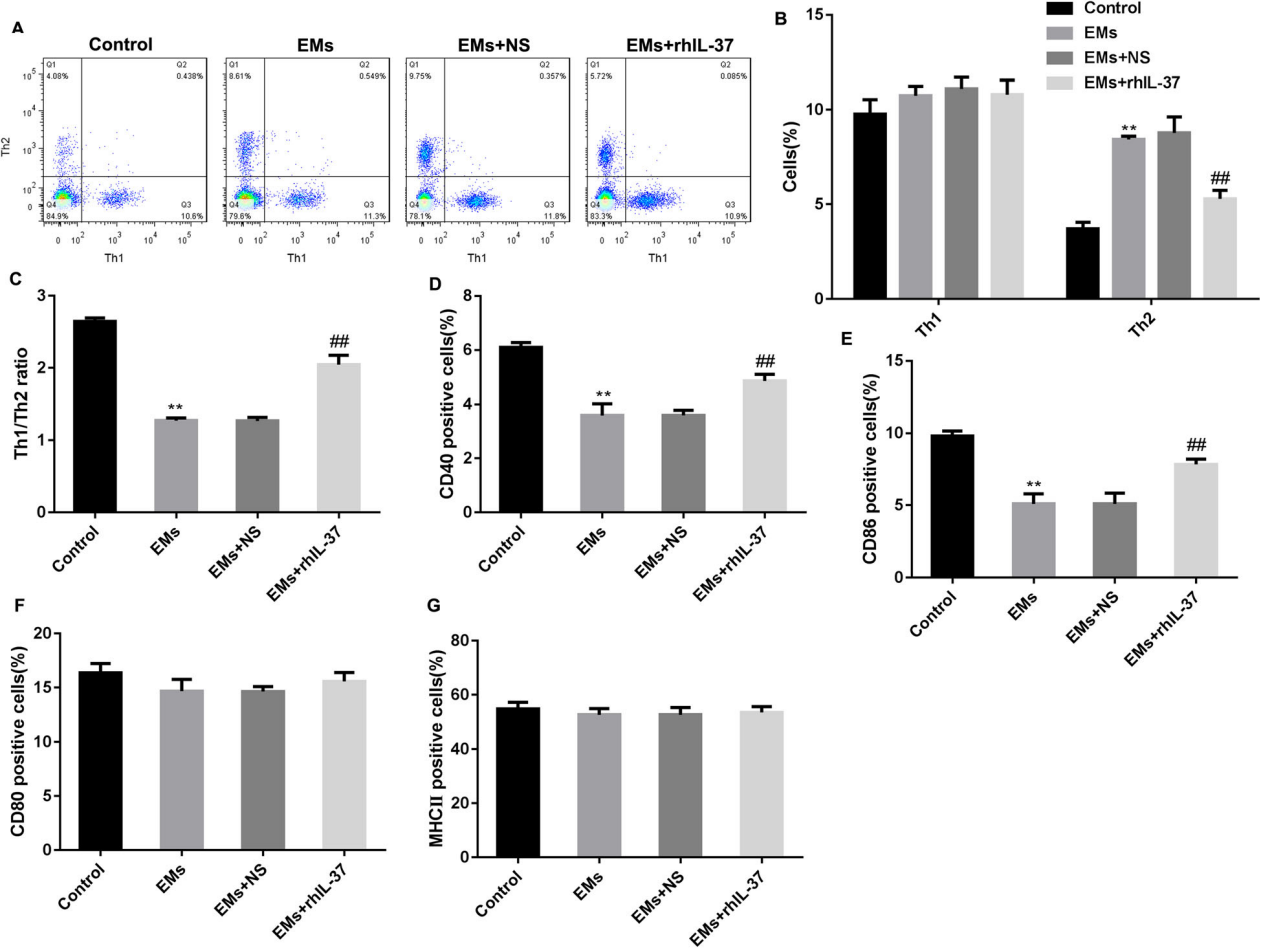
rhIL-37 increased the serum Th1/Th2 ratio and promoted DCs to mature in the endometriosis mouse model. At 24 h after the last rhIL-37 administration, (**A**-**C**) the percentages of Th1 and Th2 cells, and the ratio of Th1/Th2 cells in serum were analyzed using flow cytometry; (**D**-**G**) the percentages of CD40-, CD80-, CD86-, and MHC II-positive DCs in serum were determined using flow cytometry. *N =* 6. **P < 0.01 compared with Control, and ^##^*P* < 0.01 compared with EMs

Furthermore, we detected the percentages of Th1 and Th2 cells, and percentages of surface maturation markers-positive DCs, including CD40-positive DCs, CD86-potitive DCs, MHC-II-positive DCs, and CD80-positive DCs, in the blood of mice. The results showed that there was no significant difference in the proportion of Th1 cells. The proportion of Th2 cells was significantly upregulated, while the ratio of Th1/Th2 cells was downregulated in the mice with endometriosis, which were partly recused by rhIL-37 treatment (Fig. 2A-C). The percentages of CD40-positive DCs and CD86-positive DCs were declined in the mice with endometriosis, but rhIL-37 treatment increased the percentage of them (Fig. 2D-E, and Supplementary Fig. 1). Besides, there was no significant difference in the percentages of CD80-positive DCs and MCH II-positive DCs (Fig. 2F-G, and Supplementary Fig. 1). Overall, above data indicated that rhIL-37 induced the maturation of DCs, increased Th1/Th2 percentages, and improved endometriosis.

### rhIL-37 upregulated Th1/Th2 ratio through inducing DCs to mature

**Fig. 3:**
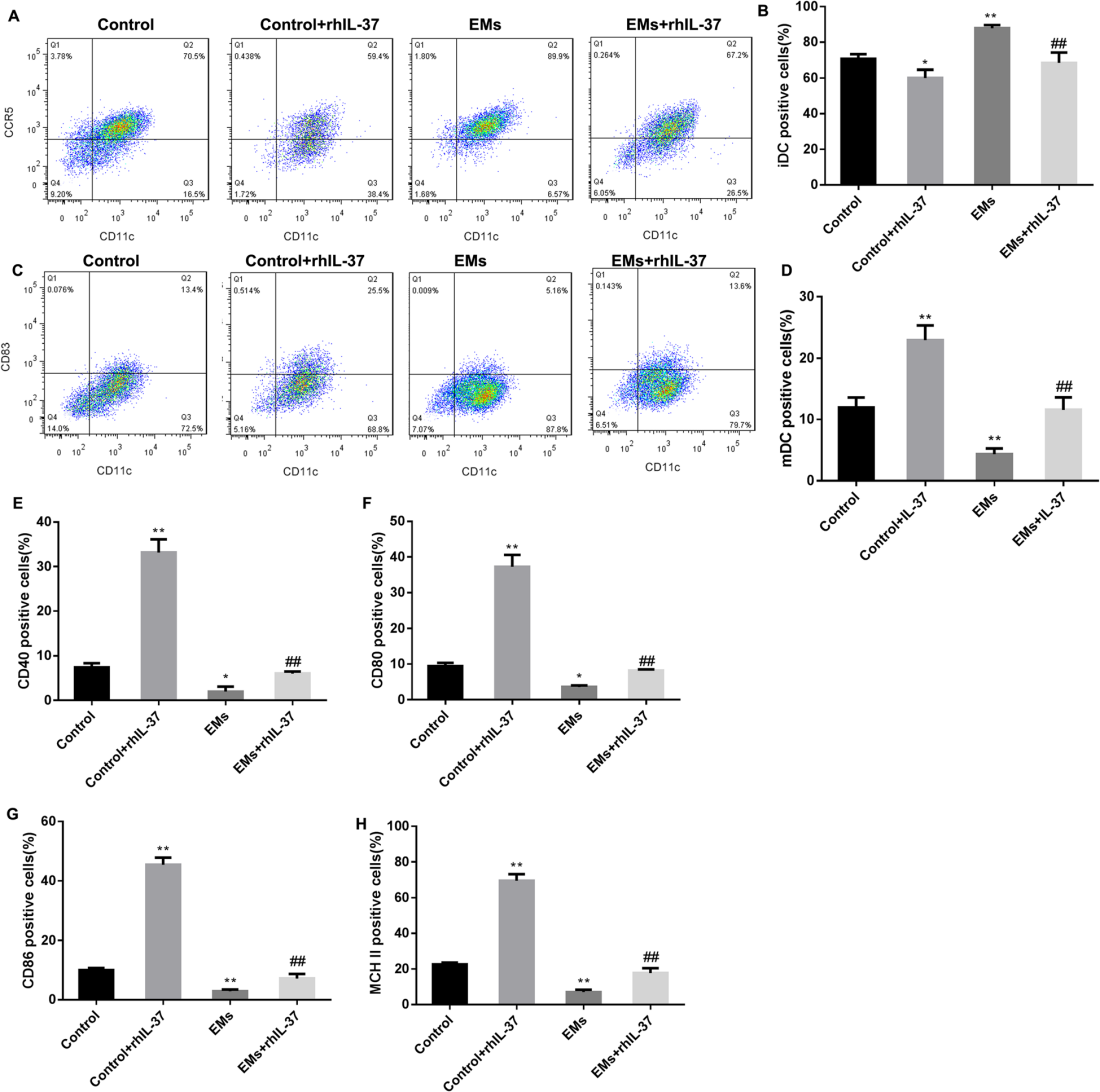
rhIL-37 promoted the maturation of DCs. The control-DCs and EMs-DCs were separated, and were then treated with rhIL-37. (**A**-**H**) The percentages of iDCs, mDCs, and the percentages of CD40-, CD80-, CD86-, and MHC II-positive DCs were determined using flow cytometry. *N =* 3. **P < 0.01 compared with Control, and ^##^*P* < 0.01 compared with EMs

In order to explore whether rhIL-37 improves endometriosis through increasing Th1/Th2 ratio by inducing DCs maturation, we separated CD4^+^T cells successfully from the peripheral blood of healthy mice (Supplementary Fig. 2), and DCs from the peripheral blood of healthy mice (control-DCs) and mouse with endometriosis (EMs-DCs) (Supplementary Fig. 3). Control-DCs and EMs-DCs were treated with 100 ng/mL rhIL-37 for 24 h. Our results displayed that rhIL-37 treatment could decline the percentage of iDCs in both control-DCs and EMs-DCs, and the percentage of iDCs was higher in EMs-DCs than that in control-DCs (Fig. 3A and B). Oppositely, the percentage of mDCs was lower in EMs-DCs than that in control-DCs, rhIL-37 treatment could promote the maturation of DCs (Fig. 3C and D). The images of iDCs and mDCs were shown as in Supplementary Fig. 4. Subsequently, LPS was used to stimulate the rhIL-37-treated control-DCs and EMs-DCs for another 2 days. The percentages of CD40-, CD80, CD86-, and MCH II-positive DCs were significantly lower in EMs-DCs than that in control-DCs. In both control-DCs and EMs-DCs, rhIL-37 treatment increased the percentages of CD40-, CD80, CD86-, and MCH II-positive DCs (Fig. 3E-H, and Supplementary Fig. 5). Above results suggested that rhIL-37 could contribute to the maturation of DCs.

**Fig. 4:**
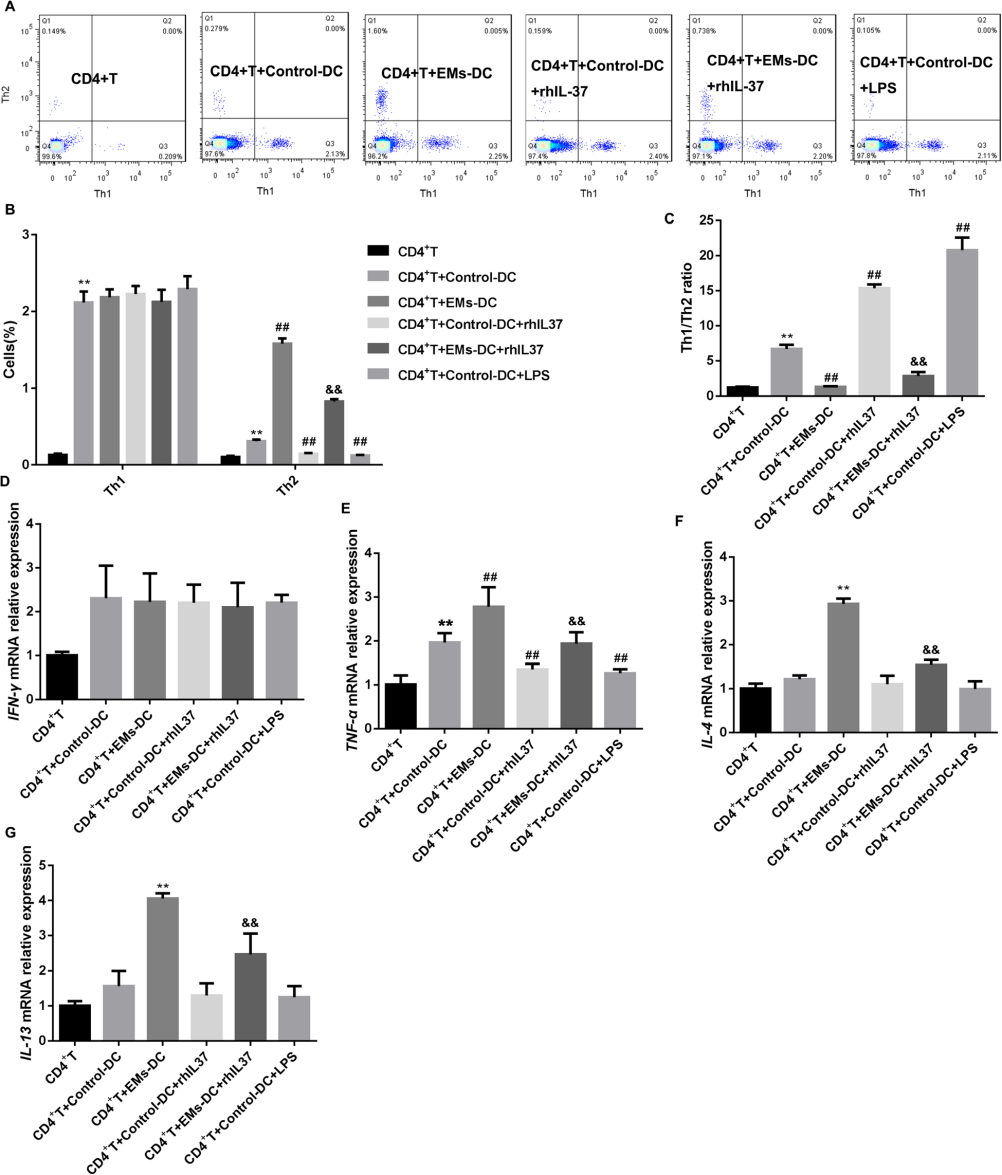
rhIL-37 increased the ratio of Th1/Th2 via inducing EMs-DCs to mature. The control-DCs and EMs-DCs were co-cultured with CD4^+^T cells. At the same time, the DCs were treated with or without rhIL-37 and LPS. (**A**-**C**) The percentages of Th1 and Th2 cells, and the ratio of Th1/Th2 cells in CD4^+^T cells was analyzed by flow cytometry; (**D**-**G**) the expression levels of IFN-γ mRNA, TNF-α mRNA, IL-4 mRNA, and IL-13 mRNA in CD4^+^T cells were measured by qRT-PCR. *N =* 3. **P < 0.01 compared with CD4^+^T, ^##^*P* < 0.01 CD4^+^T + Control-DC, and ^&&^*P* < compared with CD4^+^T + EMs-DCs

Next, we constructed a co-culture system of DCs with CD4^+^T cells. CD4^+^T were co-cultured with control-DCs, EMs-DCs, rhIL-37-treated control DCs, rhIL-37-treated EMs-DCs, and LPS-treated DCs, respectively. Our data showed that DCs promoted Th1 differentiation, which was not related to whether the DCs was control-DCs or EMs-DCs, and whether the DCs accepted with rhIL-37 treatment. Compared with control-DCs, EMs-DCs significantly promoted Th2 differentiation and downregulated Th1/Th2 ratio, which were partly reversed by rhIL-37 treatment (Fig. 4A-C). Moreover, our results also demonstrated that the expression of IFN-γ, TNF-α, IL-4, and IL-13 mRNAs was facilitated in the CD4^+^T cells co-cultured with DCs. Compared with control-DCs, EMs-DCs boosted the expression of TNF-α, IL-4 and IL-13 mRNAs in CD4^+^T cell. Importantly, rhIL-37 and LPS treatment could notably inhibit the expression of TNF-α, IL-4, and IL-13 mRNAs (Fig. 4D-G). In conclusion, rhIL-37 inhibited Th2 differentiation and increased Th1/Th2 ratio through inducing DCs to mature.

### rhIL-37 upregulated Th1/Th2 ratio by suppressing the production of IL-4 in DCs

**Fig. 5:**
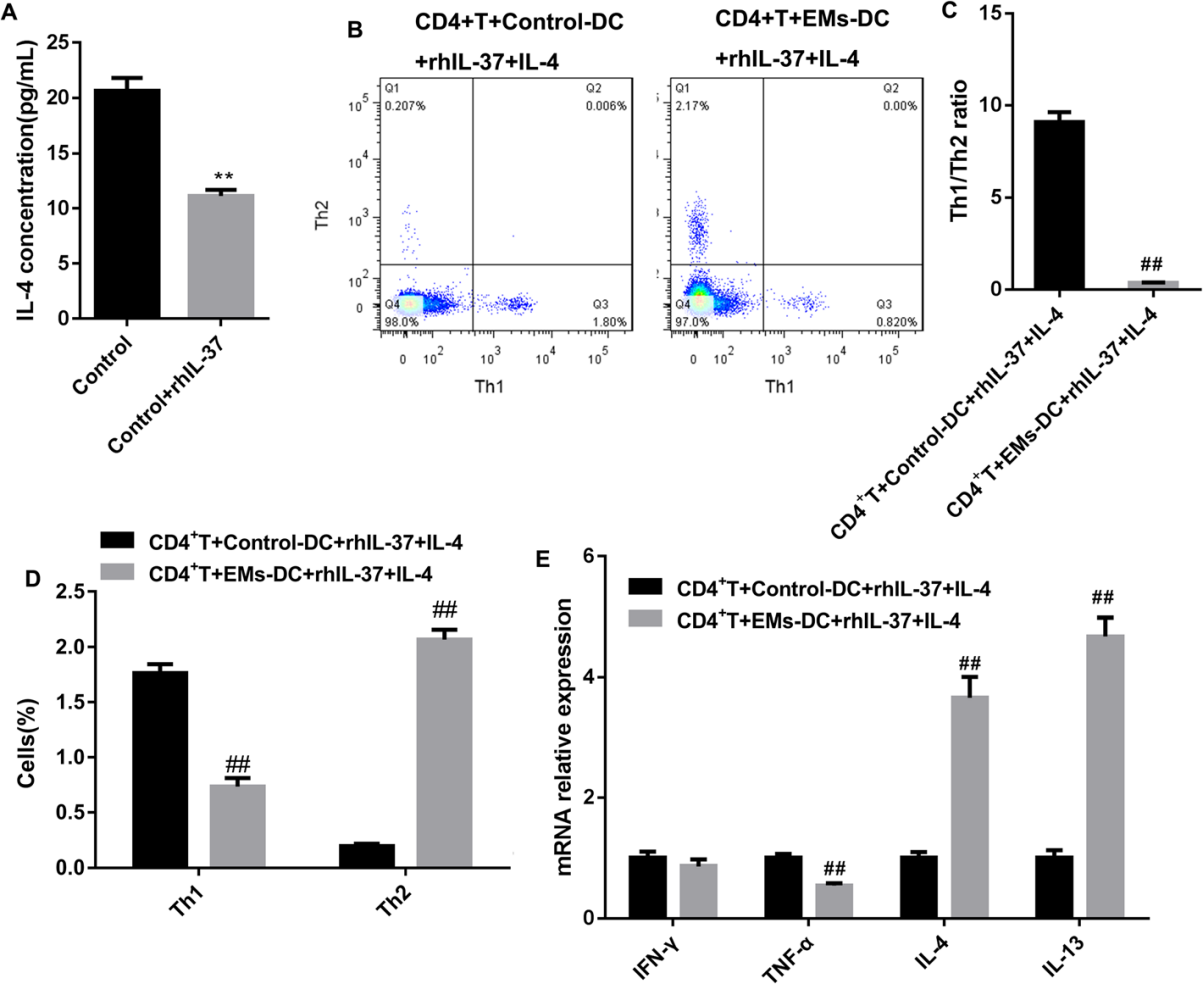
IL-4 mediated the promotion of EMs-DCs to Th2 differentiation. (**A**) Control-DCs were treated with rhIL-37, and then the production of IL-4 was examined using ELISA. *N =* 3. **P < 0.01 compared with Control. (**B**-**D**) The percentages of Th1 and Th2 cells, and the ratio of Th1/Th2 cells in CD4^+^T cells was analyzed by flow cytometry; (**E**) the expression levels of IFN-γ mRNA, TNF-α mRNA, IL-4 mRNA, and IL-13 mRNA in CD4^+^T cells were measured by qRT-PCR. *N =* 3. ^##^*P* < 0.01 compared with CD4^+^T + Control-DCs + rhIL-37 + IL-4

Subsequently, we explored the mechanism of Th1/Th2 ratio increasing induced by DCs. IL-4 is a main factor that induces the differentiation of Th2 cells. Here, rhIL-37 stimulation significantly suppressed the production of IL-4 in control-DCs (Fig. 5A). Here, to ensure whether rhIL-37-treated DCs increases Th1/Th2 ratio via regulating IL-4, we used IL-4 combined with rhIL-37 to treat control-DCs and EMs-DCs. The flow cytometry results revealed that Th1/Th2 ratio was significantly lower in the CD4^+^T cells co-cultured with EMs-DCs than that in the CD4^+^T cell co-cultured with control-DCs (Fig. 5B and C). Besides, compared with CD4^+^T cell co-cultured with control-DCs, the proportion of Th1 and expression of TNF-α were decreased, while the proportion of Th2 and expression of IL-4 and IL-13 were increased in the CD4^+^T cells co-cultured with EMs-DCs (Fig. 5D and E). Summary, rhIL-37 could increase Th1/Th2 ratio via inhibiting the production of IL-4 in DCs.

### rhIL-37 promoted DCs to mature via inhibiting the phosphorylation of STAT3

**Fig. 6:**
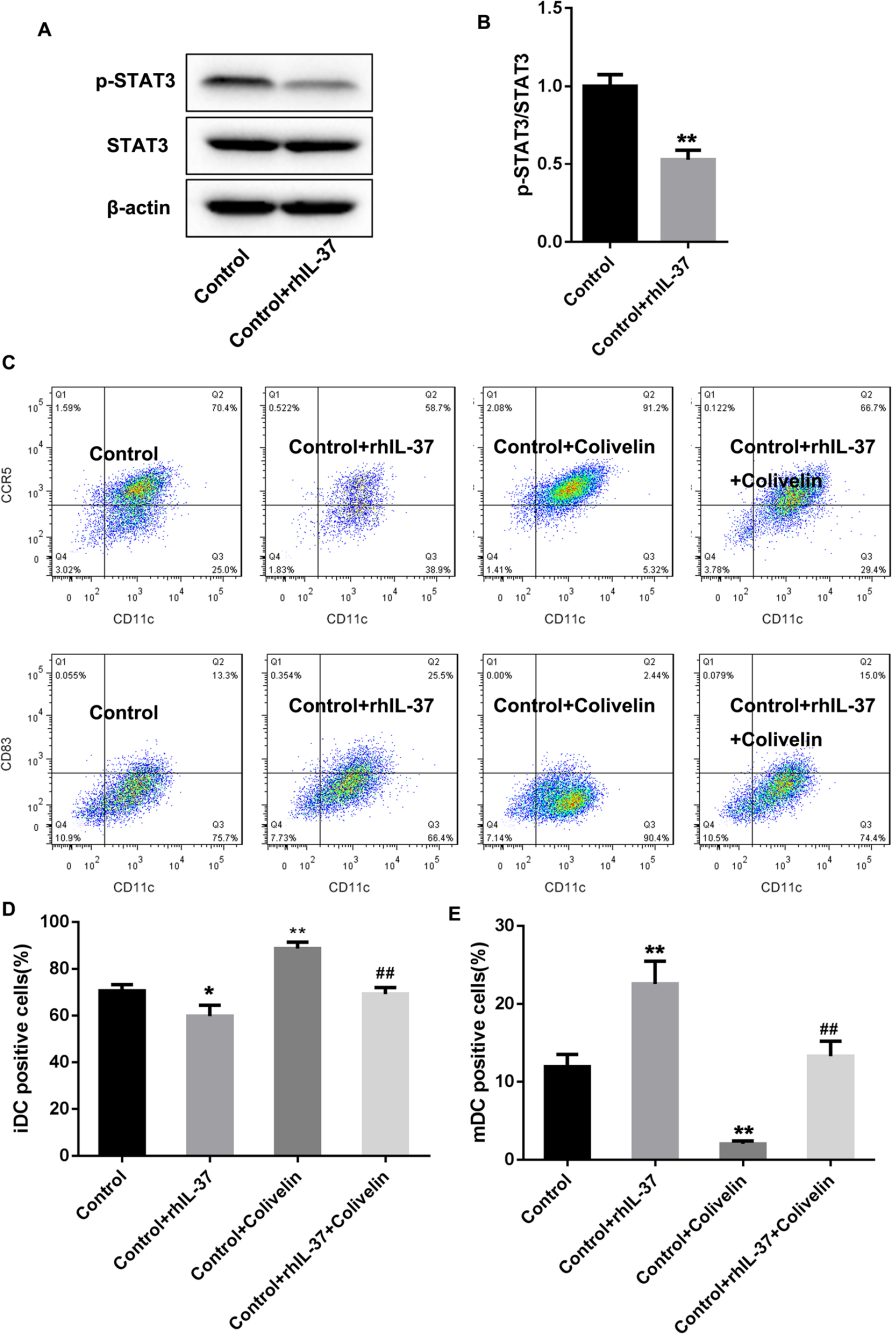
rhIL-37-induced the maturation of DCs was reversed by activation of STAT3. (**A** and **B**) Control-DCs were treated with rhIL-37, and then the expression of STAT3 and STAT3 phosphorylation were detected using western blotting assay. *N =* 3. **P < 0.01 compared with Control. (**C**-**E**) The percentages of iDCs and mDCs were measured by flow cytometry. *N =* 3. *P < 0.05 and **P < 0.01 compared with Control. ^##^*P* < 0.01 compared with Control + rhIL-37

**Fig. 7:**
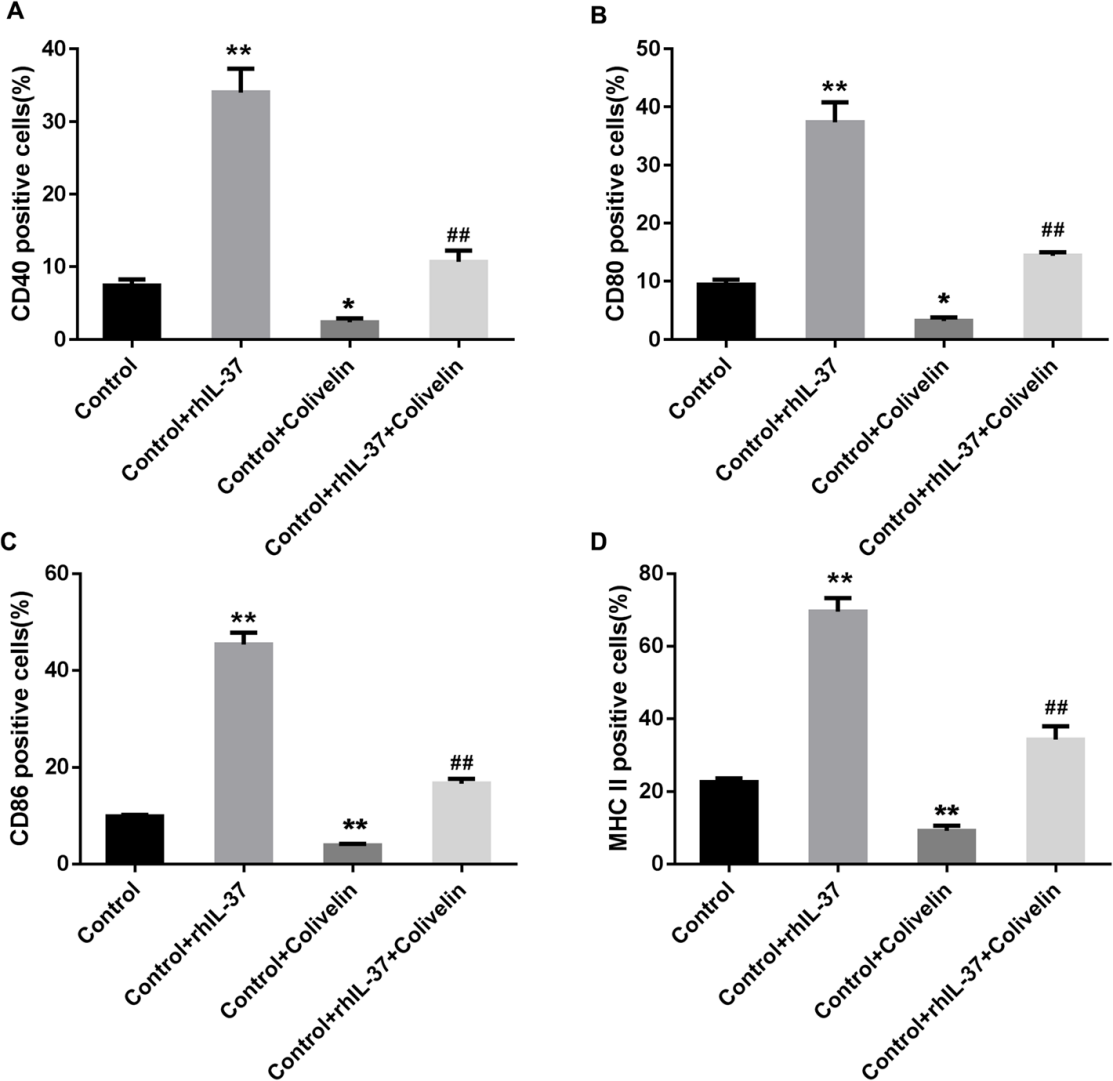
rhIL-37-induced the increasing of CD40, CD80, CD86, and MHC II in DCs was rescued by activation of STAT3. (A-D) The percentages of CD40-, CD80-, CD86-, and MHC II-positive DCs were determined using flow cytometry. *N =* 3. **P < 0.01 compared with Control, and ^##^*P* < 0.01 compared with Control + rhIL-37

However, how rhIL-37 induces the maturation of DCs remains unclear. Our results indicated that the phosphorylation level of STAT3 was notably downregulated in the control-DCs by rhIL-37 treatment (Fig. 6A and B). Then, control-DCs were pre-treated with Colivelin, an activator of STAT3, followed by rhIL-37 treatment, and these control-DCs were co-cultured with CD4^+^T cells. Colivelin pre-treated DCs notably upregulated the percentage of iDCs and downregulated the percentage of mDCs. The rhIL-37-treated control-DCs-induced downregulation in the percentage of iDCs and upregulation in the percentage of mDCs was markedly recused by rhIL-37 treatment (Fig. 6C-E). Furthermore, Colivelin treatment effectively downregulated CD40-, CD80-, CD86-, and MHC II-positive DCs percentage. Meantime, rhIL-37-induced upregulation in the percentages of CD40-, CD80-, CD86-, and MHC II-positive DCs were reversed by the phosphorylation of STAT3 (Fig. 7A-D, and Supplementary Fig. 6). In summary, rhIL-37 induced DCs to mature through inhibiting the phosphorylation of STAT3.

## Discussion

According to the study of Cavalli et al., IL-37 is expressed in several human tissues and cell lines. Among immune cells, IL-37 is expressed in circulating monocytes and tissues macrophages, DCs, tonsillar B cells, and plasma cells [16]. Different with other members of IL-1 family, IL-37 has been proved to be an anti-inflammation cytokine in numerous inflammatory disorders, including endometriosis [19]. However, the action mechanism of rhIL-37 in endometriosis development still is not clear. In this study, our data showed that rhIL-37 treatment could effectively inhibit the development of ectopic lesions in the mice with endometriosis. A recent study reported that IL-37 plays an anti-tumor immunity role in the progression of hepatocellular carcinoma through promoting the recruitment of DCs and inducing the activation of DCs [20]. However, in another study, IL-37 was proved to suppress the maturation of DCs through targeting IL-1R8/ toll-like receptor 4/NF-κB signaling pathway [21]. CD40, CD80, CD86, and MHC II are the surface markers of mature DCs [22]. These studies suggested that DCs is a target of IL-37. Here, rhIL-37 treatment significantly increased the proportion of CD40-, CD80-, CD86-, and MHC II-positive DCs in the blood of the mice with endometriosis, suggesting that rhIL-37 promoted DCs maturation in endometriosis. Moreover, a previous study revealed that IL-37 notably inhibits the differentiation of Th2 and Th17, and suppresses the expression of effector cytokines like IL-4, IL-5, and IL-6. However, IL-37 treatment has no significant effect on Th1 and Treg cells differentiation, and the expression of IFN-γ and IL-10 [23]. It is not clear that how about the effect of rhIL-37 on Th1 and Th2 differentiation in endometriosis. In our present study, we found that rhIL-37 has no effect on Th1 differentiation, but inhibited Th2 differentiation in endometriosis mouse model. Importantly, our results showed that rhIL-37 increasing Th1/Th2 ratio through inducing the maturation of DCs.

Endometriosis is a chronic and pro-inflammatory disease. T cells, NK cells, and other immune cells play a crucial role in the development of the disease. Immune cells, pro-inflammatory cytokines and adhesion molecules provide suitable conditions for the differentiation, adhesion, proliferation and survival of ectopic endometrial cells [24–26]. Söhngen at al. indicated that the T cell-deficient mice can be used to establish endometriosis mouse model without additional clearance the B cell, suggesting the important role of T cells in endometriosis development [27]. The number of T cells was markedly increased in the peritoneal fluid of the patients with endometriosis at early stage. Subsequently, with the development of endometriosis, apoptotic rate of T cells was increasing [28]. The percentage of Th1 cells was lower in the endometriosis tissues than that in the endometrial tissues [29]. Besides, Chen et al. demonstrated that the Th1 cell-related cytokine was lower, and Th2 cell-related cytokine was higher in the endometriosis tissues in comparison with the endometrial tissues [30]. Due to rhIL-37 has no effect on the differentiation of Th1, we explored only the pathway for rhIL-37 inhibiting Th2 differentiation. IL-4 is a crucial inducer for the differentiation of Th2 cells [31]. The inhibitory effect of rhIL-37 on IL-4 expression in DCs was found in our study. Furthermore, we proved that rhIL-37 increased Th1/Th2 ratio through inhibiting the production of IL-4 in DCs.

STAT3 is a transcriptional factor. It was proved that the hyperactivation of STAT3 may resulted in the occurrence of autoimmunity and immunodeficiency through regulating immune cells [32]. In tumor, the hyperactivation of STAT3 has been proved to suppress the maturation of bone marrow-derived DCs [33]. However, in endometriosis development, the effect of STAT3 on DCs maturation remains unclear. Kim et al. demonstrated that the phosphorylation level of STAT3 is significantly higher in the endometriosis tissues than that in the normal endometrial tissues [34]. In addition, it was reported that extracellular IL-37 can regulate the downstream STAT3 signaling [35]. These researches suggested that rhIL-37 may affect DCs maturation via regulating the phosphorylation of STAT3. In our present study, our data revealed that activation of STAT3 could effectively reverse rhIL-37-induced the maturation of DCs.

## Conclusions

Overall, our data demonstrated that rhIL-37 markedly inhibited the development of endometriosis via increasing the ratio of Th1/Th2 cells by inhibiting the production of IL-4 in DCs and promoting the maturation of DCs. Mechanismly, rhIL-37 promoted DCs maturation through suppressing the phosphorylation of STAT3. Our research may provide a novel therapeutic idea for endometriosis.

## Supplementary Information

**Additional file 1: Supplementary figure 1**. Detection of the mature DCs percentage. At 24 hours after the last rhIL-37 administration, the percentages of CD40-, CD80-, CD86-, and MHC II-positive DCs in serum were determined using flow cytometry. *N* = 3.

**Additional file 2: Supplementary figure 2**. Analysis of the CD4^+^T cells. Flow cytometry was used to isolate CD4^+^T cells from the peripheral blood of healthy mice. *N =* 3.

**Additional file 3: Supplementary figure 3**. Analysis of the DCs cells. Flow cytometry was used to isolate DCs from the peripheral blood of healthy mice and endometriosis mouse model. *N =* 3.

**Additional file 4: Supplementary figure 4**. Detection of the iDCs and mDCs percentages. The control-DCs and EMs-DCs were separated, and were then treated with rhIL-37. The percentages of iDCs, mDCs were determined using flow cytometry. *N =* 3.

**Additional file 5: Supplementary figure 5**. Analysis of the maturation of DCs. DCs and EMs-DCs were separated, and were then treated with rhIL-37, and then the percentages of CD40-, CD80-, CD86-, and MHC II-positive DCs were determined using flow cytometry. *N =* 3.

**Additional file 6: Supplementary figure 6**. Analysis of the maturation of DCs. The percentages of CD40-, CD80-, CD86-, and MHC II-positive DCs were determined using flow cytometry. *N =* 3.

**Additional file 7: Supplementary Fig. 7**. The drug administration, sampling, and sacrifice process to the mice.

## Data Availability

The datasets used and/or analysed during the current study are available from the corresponding author on reasonable request.
