# Supplementary figures and images for: Recombinant human IL-37 inhibited endometriosis development in a mouse model through increasing Th1/Th2 ratio by inducing the maturation of dendritic cells
Source: Reprod Biol Endocrinol. 2021 Aug 24;19:128. doi: 10.1186/s12958-021-00811-3 (PMC8383354; doi:10.1186/s12958-021-00811-3)

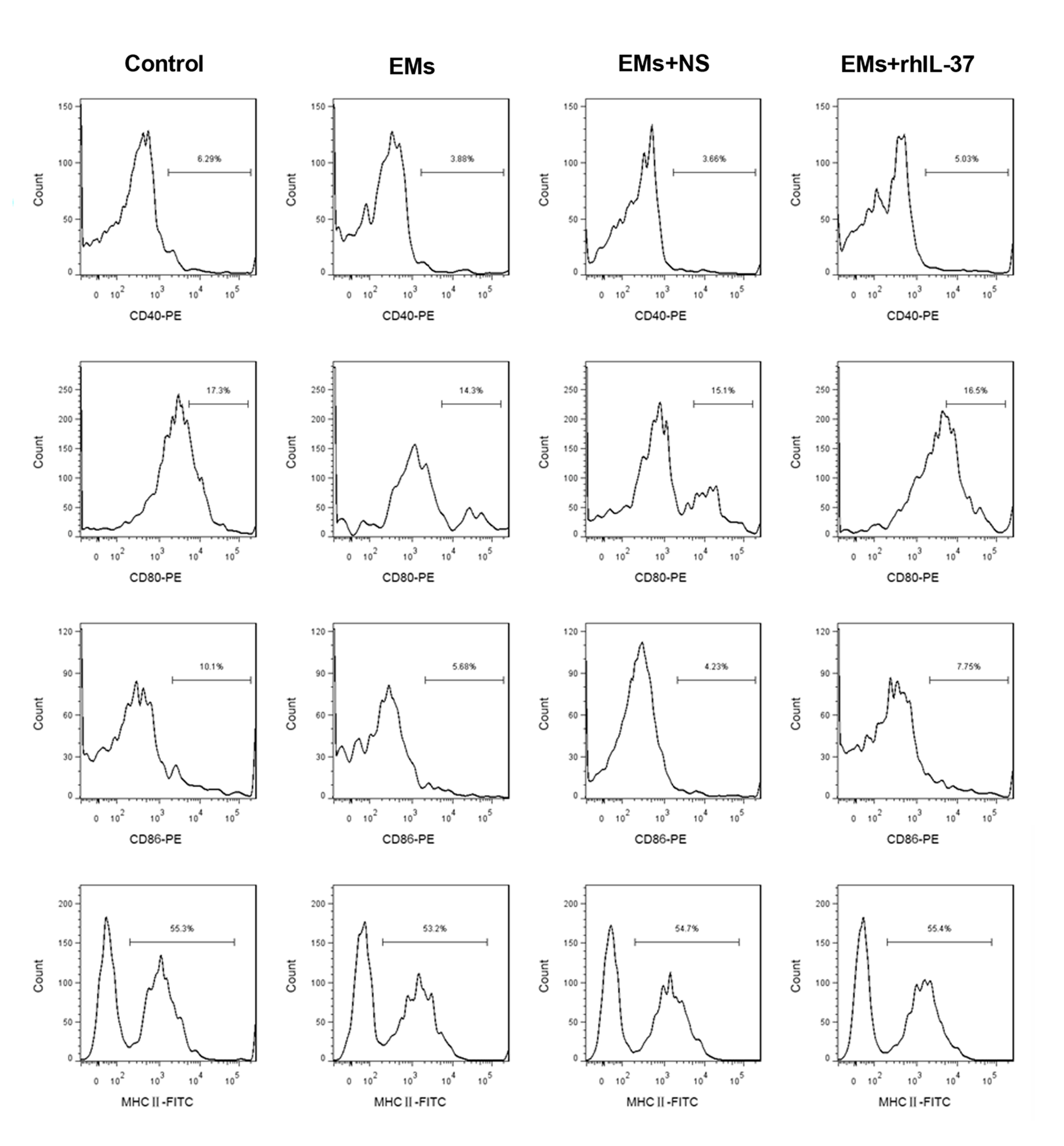

Supplement: Supplementary file 1 — Additional file 1: Supplementary figure 1. Detection of the mature DCs percentage. At 24 hours after the last rhIL-37 administration, the percentages of CD40-, CD80-, CD86-, and MHC II-positive DCs in serum were determined using flow cytometry. N = 3. [file 12958_2021_811_MOESM1_ESM.tif]

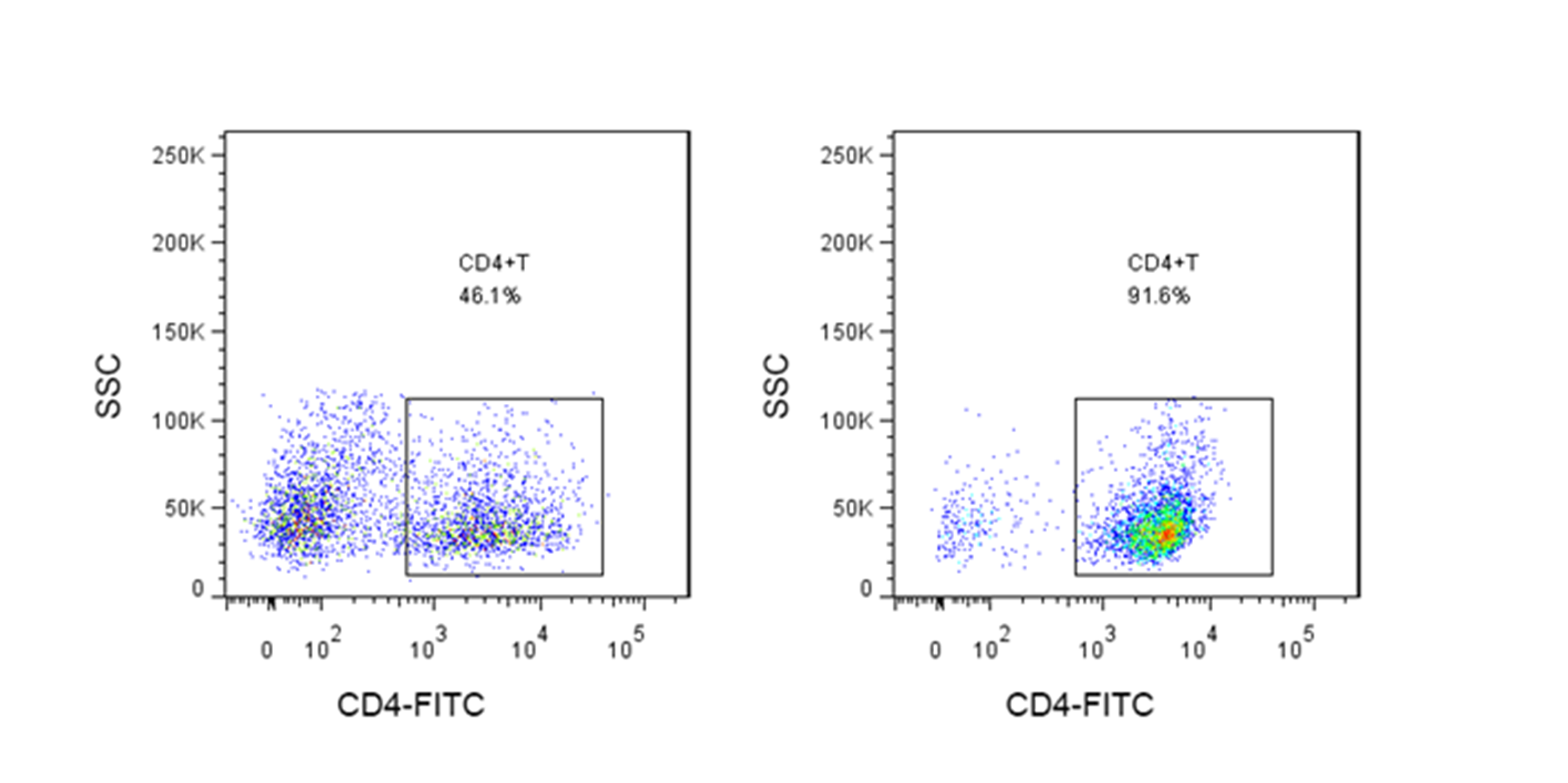

Supplement: Supplementary file 2 — Additional file 2: Supplementary figure 2. Analysis of the CD4+T cells. Flow cytometry was used to isolate CD4+T cells from the peripheral blood of healthy mice. N = 3. [file 12958_2021_811_MOESM2_ESM.tif]

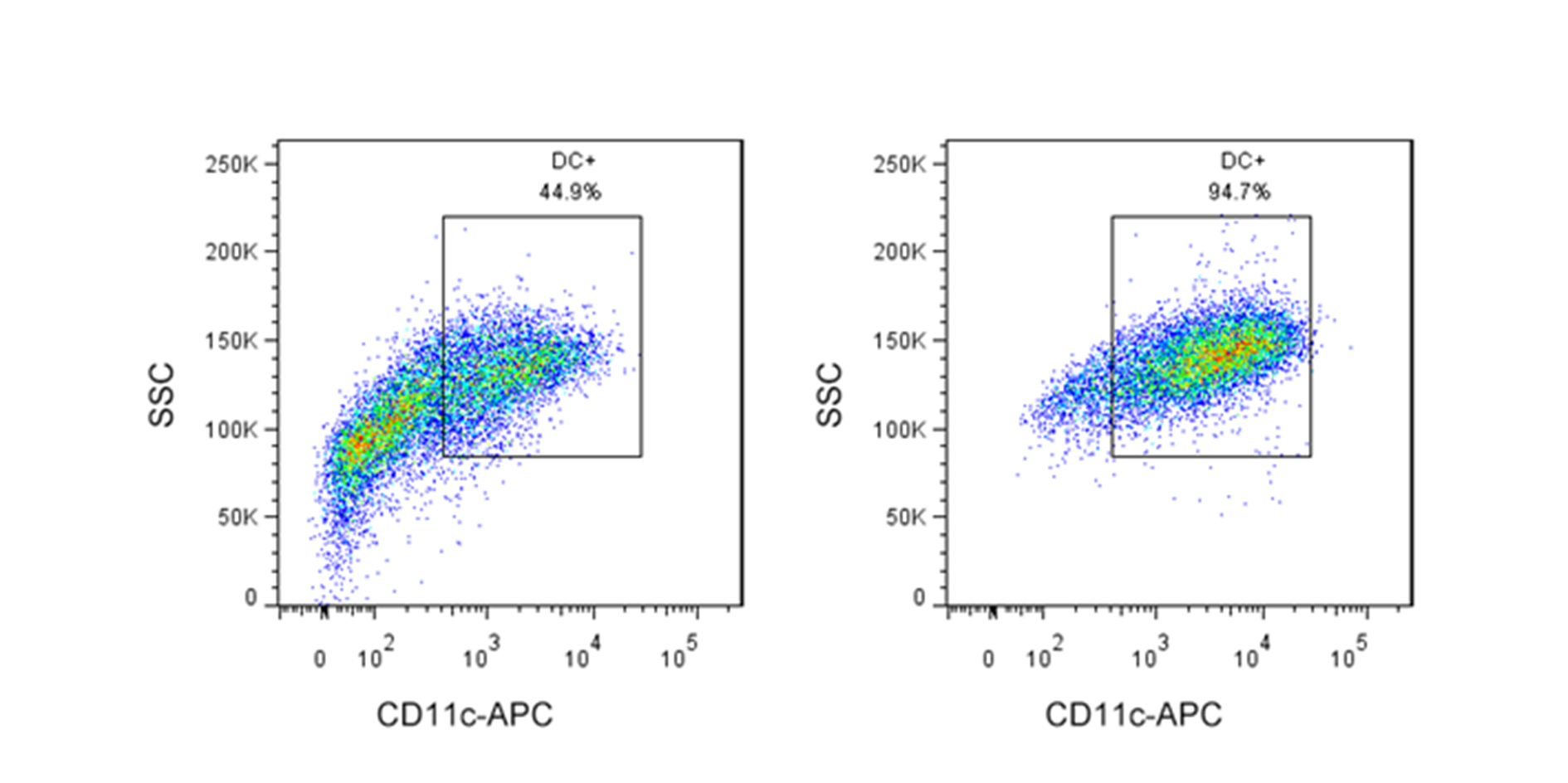

Supplement: Supplementary file 3 — Additional file 3: Supplementary figure 3. Analysis of the DCs cells. Flow cytometry was used to isolate DCs from the peripheral blood of healthy mice and endometriosis mouse model. N = 3. [file 12958_2021_811_MOESM3_ESM.tif]

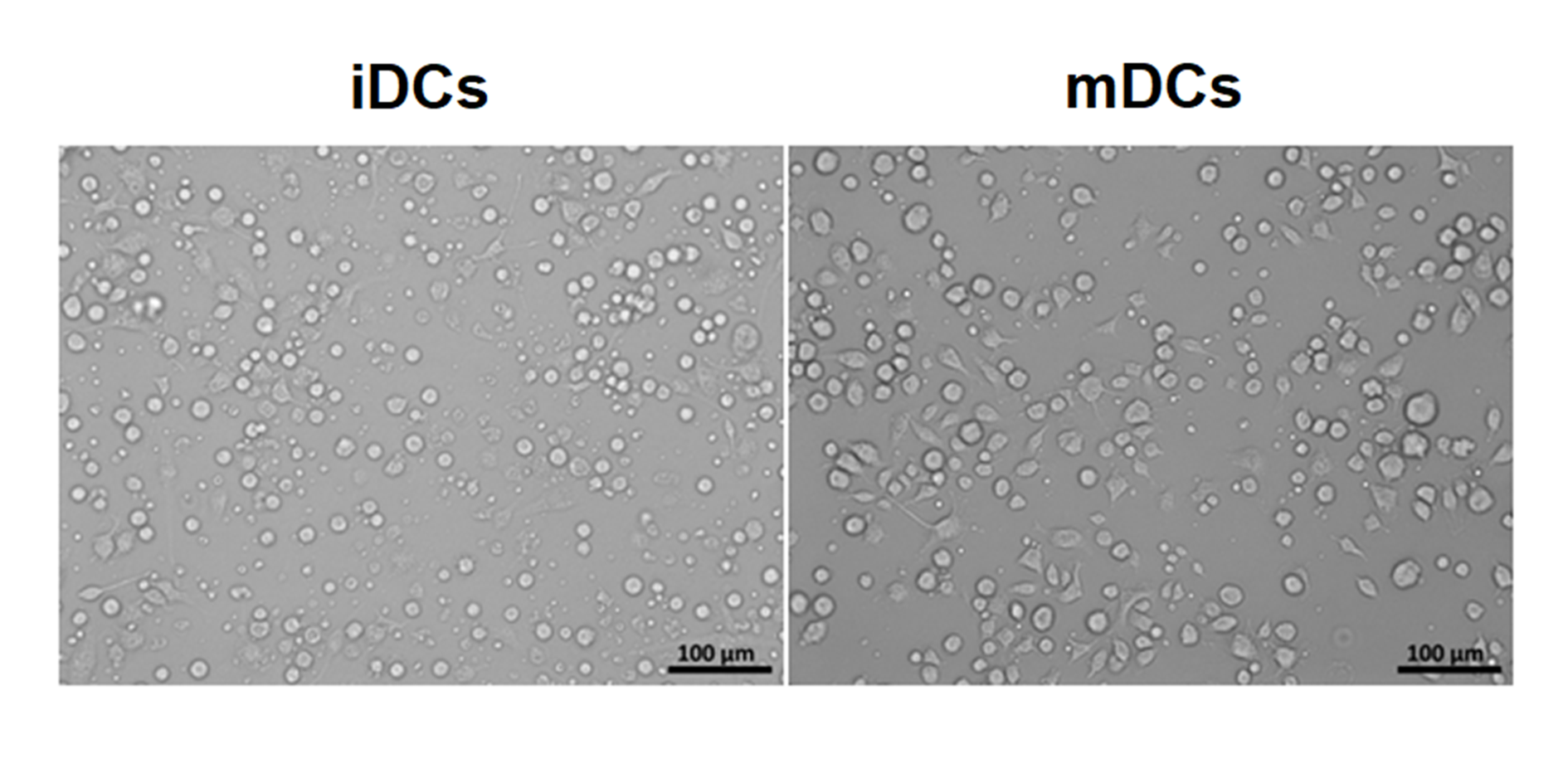

Supplement: Supplementary file 4 — Additional file 4: Supplementary figure 4. Detection of the iDCs and mDCs percentages. The control-DCs and EMs-DCs were separated, and were then treated with rhIL-37. The percentages of iDCs, mDCs were determined using flow cytometry. N = 3. [file 12958_2021_811_MOESM4_ESM.tif]

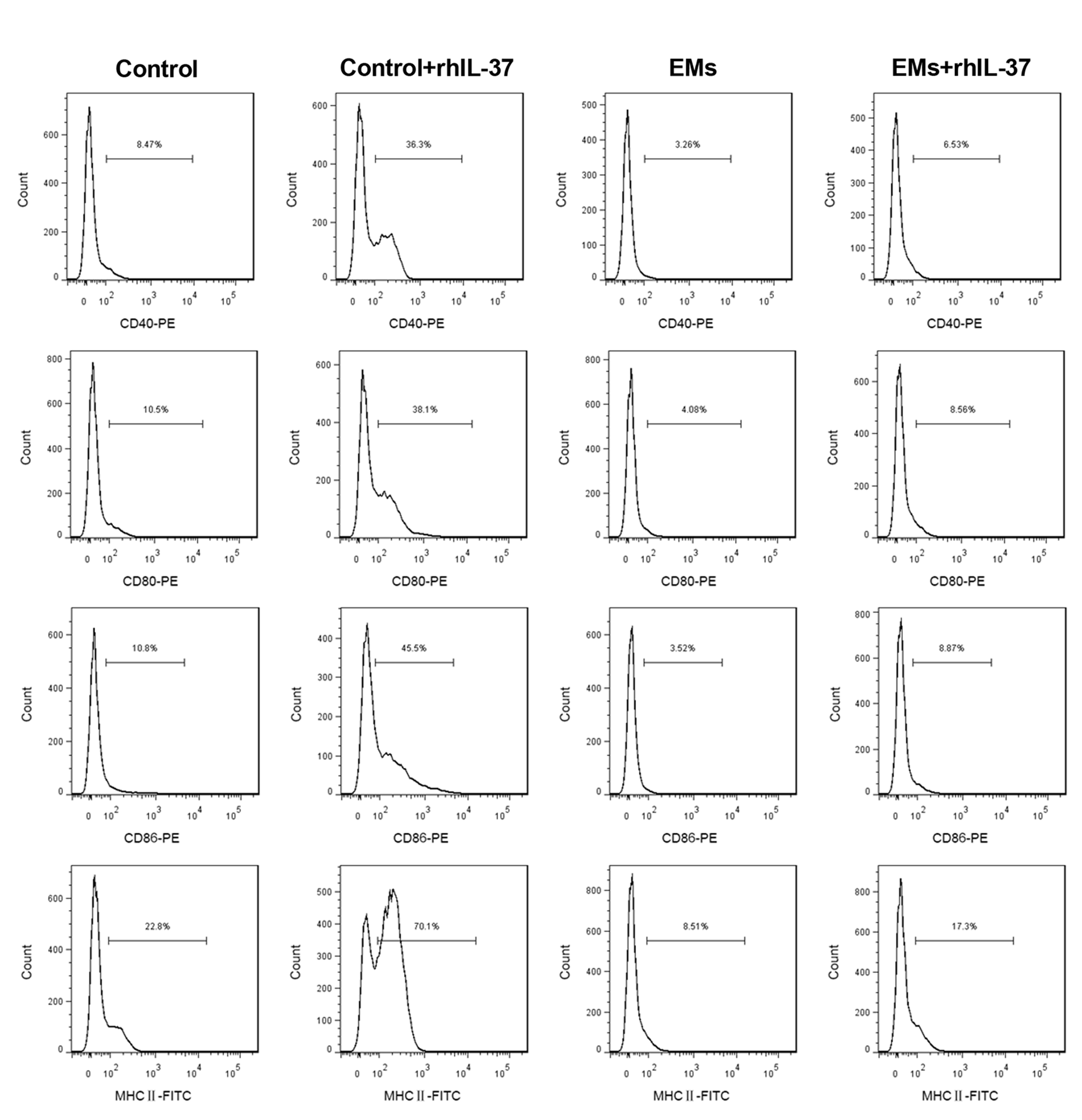

Supplement: Supplementary file 5 — Additional file 5: Supplementary figure 5. Analysis of the maturation of DCs. DCs and EMs-DCs were separated, and were then treated with rhIL-37, and then the percentages of CD40-, CD80-, CD86-, and MHC II-positive DCs were determined using flow cytometry. N = 3. [file 12958_2021_811_MOESM5_ESM.tif]

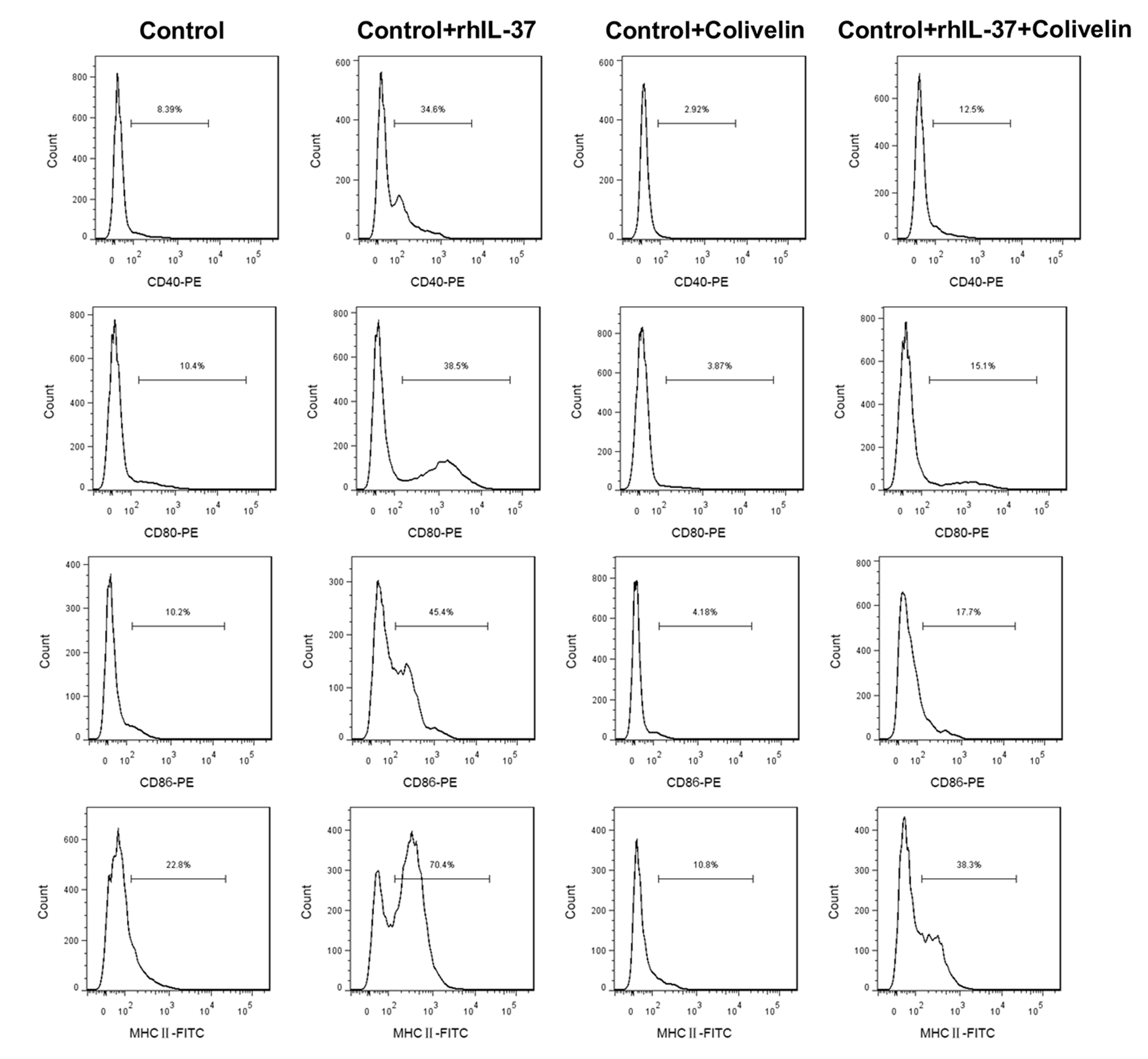

Supplement: Supplementary file 6 — Additional file 6: Supplementary figure 6. Analysis of the maturation of DCs. The percentages of CD40-, CD80-, CD86-, and MHC II-positive DCs were determined using flow cytometry. N = 3. [file 12958_2021_811_MOESM6_ESM.tif]

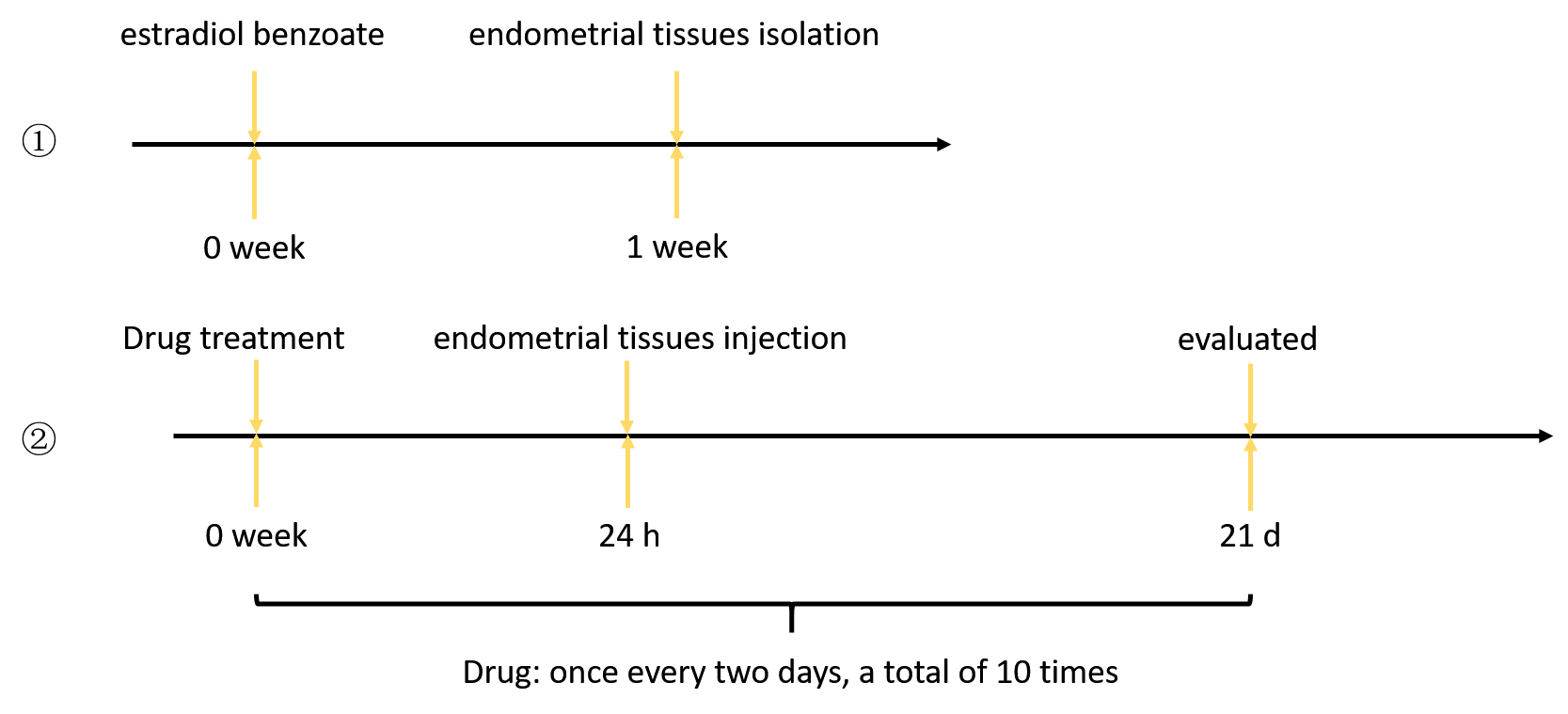

Supplement: Supplementary file 7 — Additional file 7: Supplementary Fig. 7. The drug administration, sampling, and sacrifice process to the mice. [file 12958_2021_811_MOESM7_ESM.tif]
